# Investigating the Prospective Relationship Between Weight Loss Behaviours and Sleep in Adolescents From the Growing Up in Ireland Cohort

**DOI:** 10.1002/erv.70045

**Published:** 2025-10-25

**Authors:** Marie‐Christine Opitz, Sarah Cooney, Nora Trompeter, Sylvane Desrivières, Nadia Micali, Ulrike Schmidt, Helen Sharpe

**Affiliations:** ^1^ Department of Clinical Psychology School of Health in Social Sciences University of Edinburgh Edinburgh Scotland; ^2^ School of Psychology University College Dublin Dublin Ireland; ^3^ Great Ormond Street Institute of Child Health University College London London UK; ^4^ Social Genetic and Developmental Psychiatry Centre Institute of Psychiatry Psychology and Neuroscience King's College London London UK; ^5^ Centre for Research in Eating and Weight Disorders Institute of Psychiatry Psychology and Neuroscience King's College London London UK; ^6^ South London and Maudsley NHS Foundation Trust London UK

**Keywords:** growing up in Ireland, restriction, sleep, weight loss behaviours

## Abstract

**Objective:**

Despite common bio‐behavioural mechanisms underlying maladaptive sleep and eating, little is known about their temporal associations. The present study aimed to assess the longitudinal relationship between weight loss behaviours (age 13) and sleep (age 17/18) in adolescents (*N* = 5705) from the ’98 Growing Up in Ireland cohort.

**Method:**

Using structural equation modelling, regression models were specified and excessive online behaviours (age 17/18) were tested as a moderator, while depressive symptoms (age 13) were tested as a mediator for the prospective association.

**Results:**

Weight loss behaviours significantly predicted falling asleep at inappropriate times (*β* = 0.16, SE = 0.04, *p* < 0.001) and shorter sleep duration (*β* = 0.06, SE = 0.02, *p* < 0.001), but no other sleep behaviours. Associations with falling asleep at inappropriate times were partially mediated through depressive symptoms. Associations with shorter sleep duration were fully mediated by depressive symptoms. Excessive online behaviours did not moderate the relationship between sleep and weight loss behaviours.

**Conclusions:**

In line with clinical research, this study provides preliminary evidence for the longitudinal relationship between weight loss behaviours and shorter sleep. Further research into the causal and potentially bi‐directional relationship between sleep and disordered eating is needed to aid in preventing the exacerbation of both symptom presentations and to inform general health promotion strategies.

## Highlights

- Adolescence is a vulnerable developmental stage for the onset of sleep and eating difficulties.
- In the current Irish cohort, engaging in weight loss behaviours at age 13 was associated with shorter sleep duration at age 17/18.
- Depressive symptoms were shown to be a putative mediator for this relationship.

## 1 Introduction

High prevalence of sleep difficulties (including short sleep duration, sleep onset difficulties, and night waking) among adolescents has recently been reported in Europe (20%) (Lewien et al. 2021) and Asia (26%) (Liang et al. 2021), while the US Centres for Disease Control and Prevention identified yearly increases in the frequency of young people's self‐reported insufficient sleep (77% in 2021) (CDC 2024). This is particularly concerning as sleep problems are commonly seen as a precursor, potential risk factor for, and consequence of, a variety of mental health difficulties (Uccella et al. 2023). This includes eating disorders, which can be linked to sleep through the interplay of eating‐ and sleep‐related routines, as well as hormonal processes that are involved in both the metabolic and circadian system (Allison et al. 2016). Restrictive eating (i.e., skipping meals/snacks, daytime fasting) is a transdiagnostic eating disorder behaviour, which can be bi‐directionally linked to disruptions in circadian and diurnal appetite rhythms through biochemical feedback loops (De Young and Bottera 2022). This includes sleep onset difficulties or increased fatigue due to hunger and malnutrition (Christensen and Short 2021). Importantly, adolescence is a developmental stage associated with an increased likelihood for the onset of both disordered eating and sleep problems (Cooper et al. 2020).

To explore associations between adolescents' restrictive eating and other weight loss behaviours (a risk factor for later eating disorder onset (Neumark‐Sztainer et al. 2006)) and sleep difficulties in this vulnerable developmental stage, a previous project explored the bi‐directional relationship between sleep and weight loss behaviours in a large UK adolescent sample, utilising information from the Millennium Cohort Study (MCS) (Opitz et al. 2025). Findings from this study, including *N* = 6041 adolescents, indicated a significant relationship between weight loss behaviours (age 14) and poorer sleep quality (age 17), which was fully mediated by depressive symptoms.

The present study aimed to replicate and extend these findings by investigating the longitudinal relationship between weight loss behaviours and a variety of sleep difficulties in an Irish cohort (Growing Up in Ireland = GUI). Although Ireland has a significantly lower proportional incidence rate of eating disorders when compared to the UK (Petkova et al. 2019), these numbers are likely underestimating existing cases (Driscoll et al. 2024). Interestingly, within the latest Health Behaviour in School‐aged Children (HBSC) report, Irish 13‐ and 15‐year old adolescents reported feeling ‘too fat’ more commonly than same‐aged English adolescents (Rakić et al. 2024). The high occurrence of these concerns is alarming when considering that feeling overweight, irrespective of one's actual weight, has been shown to be a risk factor for eating disorders and maladaptive compensatory behaviours (Richmond et al. 2022).

A better understanding of the longitudinal relationship between weight loss behaviours and sleep difficulties across different cultural contexts could uniquely inform much needed prevention and early intervention approaches for sleep problems and indicators of disordered eating (Cooper et al. 2020). This includes further investigations into the mechanisms potentially moderating and mediating this association. For example, depressive symptoms are associated with both eating disorders as well as sleep difficulties (Driscoll et al. 2024; Uccella et al. 2023) and have been shown to mediate the relationship between insomnia and eating disorder symptomatology (Goel et al. 2021), as well as weight loss behaviours and sleep quality (Opitz et al. 2025). Sociocultural pressures regarding bodily appearances, including thin‐ideal internalisation, can thereby explain links between dieting behaviours and depressive symptoms considering both the effects of failed dieting attempts as well as effects of caloric deprivation (Stice and Bearman 2001), while rumination and worries associated with depressed mood can explain subsequent sleep difficulties (Slavish and Graham‐Engeland 2015). Media engagement and screen time exposure have also previously been associated with both sleep and eating concerns (Chan et al. 2021; McNicholas et al. 2009). In particular, engagement with food and weight loss‐related online content may be driven by individuals' motivations to maintain eating disorder symptoms (Tan et al. 2016), with individuals seeking out online communities for advice and support towards achieving their weight‐related goals (Mincey and Michelle Hunnicutt Hollenbaugh 2022). Thus, increased engagement with online media might impact the relationship between weight loss behaviours and sleep.

### 1.1 Research Questions

1. To what extent do weight loss behaviours (age 13) predict sleep behaviours (age 17/18), including self‐reported time‐in‐bed and self‐reported sleep duration?
2. To what extent do weight loss behaviours (age 13) predict self‐reported sleep difficulties (age 17/18), including problems getting to sleep, waking up during the night, early morning awakening, difficulties with waking up in the morning, disrupted sleep, and falling asleep at inappropriate times?
3. Do depressive symptoms (age 13) mediate the association between weight loss behaviour and sleep behaviours/problems?
4. Do excessive online behaviours (age 17/18) moderate the association between weight loss behaviours and sleep behaviours/problems?

Based on previous findings, a positive association between weight loss behaviours and sleep problems was expected, mediated through depressive symptoms. Furthermore, it was expected that engagement in weight loss behaviours would be associated with shorter time‐in‐bed and self‐reported sleep duration, again, mediated through depressive symptoms. Excessive online behaviours were expected to moderate associations between weight loss behaviours and sleep.

## 2 Methodology

As a secondary data analysis, the current project utilised data from the GUI ‘child cohort’ (also called Cohort ’98). GUI is a national longitudinal study, co‐ordinated by the Department of Children, Equality, Diversity, Integration and Youth (DCEDIY) and the Central Statistics Office (CSO) in Ireland. The present study focused primarily on wave 2 and 3, when adolescents were 13 and 17/18 years old. Only demographic information was used from wave 1. A study protocol was pre‐registered on the Open Science Framework prior to data analysis, and analysis codes uploaded once analyses were completed (https://doi.org/10.17605/OSF.IO/G2PTM). Ethical approval was sought and granted by the University of Edinburgh research ethics committee (Reference: 22‐23CLPS066).

### 2.1 Participants

Information from *N* = 8568 children was included at wave 1, *N* = 7525 participated in wave 2 and *N* = 6216 participated in wave 3. Access to the dataset (the Anonymised Microdata Files = AMFs) was granted by the Irish Social Science Data Archive (ISSDA). For present analyses, we included all participants who reported on weight loss behaviours at age 13 and sleep behaviours/problems at age 17/18, resulting in a sample of *N* = 5705 adolescents. No gender differences were identified when comparing included and excluded participants, however, significant differences were identified for household income, indicating more representation of higher income and lower representation of lower income families in the included sample.

### 2.2 Measures

#### 2.2.1 Weight Loss Behaviours

*Weight loss behaviours* were assessed at wave 2, when participants were 13 years old, based on the following items, identical to those assessed in the MCS: ‘Have you ever exercised to lose weight or to avoid gaining weight?’ (‘yes’ vs. ‘no’), ‘Have you ever eaten less food, fewer calories, or foods low in fat to lose weight or to avoid gaining weight’ (‘yes’ vs. ‘no’), and ‘Which of the following are you trying to do about your weight?’ (‘lose weight’ vs. ‘gain weight’/’stay the same weight’/’I am not trying to do anything about my weight’).

#### 2.2.2 Sleep Behaviours and Problems

All sleep behaviours and problems were solely assessed at age 17/18 (wave 3). Participants' *time‐spent‐in‐bed* was assessed through self‐reports on typical bed‐ and wake‐times, that were measured as numeric timepoints, but coded within the GUI as time categories (‘On a normal weekday, what time do you normally go to bed? (note that this may be different from the time you plan to go to sleep)’—‘9pm or earlier’, ‘9–10pm’, ‘10–11pm’, ‘11–12am’, ‘After 12am’; ‘And on a normal weekday, what time do you normally get up? (note that this may be different from the time you wake up)’—‘6am or earlier’, ‘6–7am’, ‘7–8am’, ‘8–9am’, ‘after 9am’). To calculate participants' typical time‐in‐bed, each answer category was transformed into one number (e.g., 20.5 for ‘Before 9pm’, 8.5 for ‘8:00–08:59’), and wake‐up times subtracted from bed times. Considering that these values were already pre‐categorised, we subsequently aligned time‐in‐bed categories with our previous approach and recommendations from the American Academy of Sleep Medicine for adolescents (Paruthi et al. 2016). Thus, time‐in‐bed was assessed via the following categories: ≤ 8 h, > 8–< 9 h, > 9–< 10 h, and ≥ 10 h.

In addition to time‐in‐bed, participants reported on their *sleep duration* (‘On a normal week‐night, how long do you usually sleep? Do not include time you spend awake in bed’), which was coded as whole numbers, with extreme values being subsumed in the labels ‘4 or fewer’ and ‘10 or more’.

*Sleep difficulties* were assessed with the item ‘Do you have any difficulty with sleep?’, coded in our analysis as ‘Yes, a lot of difficulty’/‘Yes, some difficulty’ versus ‘No’. For those affirming sleep difficulties, a follow‐up question asked ‘What is the nature of your sleep difficulty? [tick all that apply]’: ‘Can't get to sleep at night’, ‘I go to sleep at first but wake up during the night’, ‘I wake up too early in the morning’, ‘I find it very difficult to wake up in the morning’, ‘Sleep is regularly disrupted by someone/something else’, ‘I fall asleep at inappropriate times’, ‘Nightmares/night terrors’, ‘Sleep‐walking’. All sleep difficulties were coded as binary variables (‘yes’ vs. ‘no’).

#### 2.2.3 Depressive Symptoms

*Depressive symptoms* were assessed at age 13 (wave 2) via the Short Mood and Feelings Questionnaire (SMFQ) (Angold et al. 1995) total score, a derived variable in the GUI.

#### 2.2.4 Excessive Online Behaviour

Time‐spent‐online was assessed at age 17/18 (wave 3) using the following question: ‘How much time do you spend on each of the following activities [including being online] on a typical day (where it is your main activity at the time)? For each, please answer separately for weekdays and weekend days’. We created two variables (for weekdays and weekends), contrasting *excessive online behaviours* (‘more than 3 h’) with all other pre‐specified categories (‘none’/‘less than an hour’/‘1 up to 2 h’/‘2 up to 3 h’/‘difficult to say but at least some time every day’) to capture the distinction between potentially problematic and typical adolescent behaviours, in line with previous study findings indicating > 3 h of social media use as a potential risk factor for internalising problems (Riehm et al. 2019).

#### 2.2.5 Covariates

Relevant sociodemographic covariates were included in all analyses. These included *gender* (reported as female or male, assessed at age 9), *weight category* (GUI BMI categories, assessed at age 13), and *household income* (equivalised income quintiles by country, assessed at age 13).

### 2.3 Analyses

R version 4.3.1 was used for all analyses. The amount and patterns of item missingness for predictors and outcomes were explored using Little's MAR test and the ‘naniar’ package in R_Studio_. The response rate of the overall GUI survey was 89% at the first follow‐up assessment point (age 13), of which 81% were ‘productive’ survey participants at wave 3 (age 17/18) (Murphy et al. 2019). Current R packages cannot account for robust weighted least squares (WLSMV) estimators when pooling imputed data during the estimation of Structural Equation Modelling (SEM). As WLSMV were specified for present analyses to estimate robust standard errors, missing data on covariates were substituted, if possible, from different timepoints.

Chi‐square tests, phi coefficients (dichotomous variables) and point‐biserial correlation coefficients (continuous‐categorical associations) were used to assess associations between relevant variables. A confirmatory factor analysis (CFA) was conducted to test the factor structure of the latent ‘weight loss behaviour’ factor in the GUI. SEM was implemented using ‘lavaan’ to specify individual regression and mediation analyses for all significant longitudinal associations. Of note, mediation analyses were limited by the lack of repeated assessments of mediators (i.e., depression being assessed at the same timepoint as weight loss behaviours, online behaviours being assessed at the same timepoint as sleep). Thus, temporal precedence could not be fully established in mediation analyses. Weight loss behaviours were modelled as a latent factor, while sleep variables, mediators, and covariates were added as manifest variables. Finally, a multi‐group SEM was conducted to investigate whether excessive online behaviours moderated the relationship between weight loss behaviours and sleep. A value of *p* < 0.05 was used to determine significance. Multiple testing was accounted for via the Benjamini‐Hochberg correction with a false discovery rate of 0.05 to adjust *p*‐values.

## 3 Results

An overview of the study sample's demographics is illustrated in Table 1. The prevalence of weight loss behaviours in the GUI is presented in Table 2, and sleep behaviours and difficulties are illustrated in Table 3. The mean value for depressive symptoms was *M* = 3.81 (SD = 4.23), (‘normal’ range) with no gender differences being identified. *N* = 1198 (21%) reported being online more than 3 h on a typical weekday, and *N* = 2139 (37.5%) reported being online more than 3 h on a typical week*end* day.

**TABLE 1:** Study demographics.

Participant characteristics	*N* (%)
Gender	
Female	2916 (51.1)
Male	2789 (48.9)
IOTF BMI categories	
‘Healthy’	4299 (75.4)
Overweight	1107 (19.4)
Obese	283 (5.0)
Not reported	6 (0.1)
Household income	
‘Lower quintile’	770 (13.5)
‘second quintile’	870 (15.2)
‘Third quintile’	1000 (17.5)
‘Fourth quintile’	1270 (22.3)
‘Highest quintile’	1403 (24.6)
Not reported	392 (6.9)

**TABLE 2:** Overview of weight loss behaviours in the growing up in Ireland cohort.

Weight loss behaviour	*N* (%)
Weight loss intention	1848 (32.4)
Dieting for weight loss	1750 (30.7)
Exercise for weight loss	2634 (46.2)
Frequent self‐weighing	151 (2.6)

**TABLE 3:** Overview of sleep behaviours and difficulties in the growing up in Ireland cohort.

Sleep behaviours and difficulties	*N* (%)
Sleep duration	
4 h or fewer	39 (0.7)
5 h	106 (1.9)
6 h	360 (6.3)
7 h	1376 (24.1)
8 h	2577 (45.2)
9 h	1020 (17.9)
10 h or more	227 (4.0)
Time‐in‐bed	
8 h or less	2280 (40.0)
9 h or less	2498 (43.8)
10 h or less	794 (13.9)
More than 10 h	133 (2.3)
General sleep difficulty	1664 (29.2)
Sleep onset difficulty	1200 (21.0)
Wake after sleep onset	683 (12.0)
Early awakening	233 (4.1)
Difficulty with waking up	706 (12.4)
Sleep disruptions	147 (2.6)
Falling asleep at inappropriate times	304 (5.3)

*Note:* Participants could affirm multiple sleep difficulties.

### 3.1 Correlations Between Weight Loss Behaviours, Sleep Difficulties, Depressive Symptoms, and Online Behaviours

A full overview of all correlational associations is illustrated in the Supporting Information S1. All weight loss behaviours were significantly associated with depressive symptoms and excessive online behaviours during the week (*r* = 0.05–0.09), not the weekend. While weight loss intention and dietary restriction were significantly associated with general sleep difficulties (both *r* = 0.04), the association with exercise for weight loss was non‐significant after adjusting for multiple testing. Weight loss intention and dietary restriction were significantly associated with sleep onset latency, wake after sleep onset, and falling asleep at inappropriate times (*r* = 0.03–0.05). Exercise for weight loss was only significantly associated with falling asleep at inappropriate times (*r* = 0.05). Although significant, all effect sizes were considered small.

### 3.2 Longitudinal Associations Between Weight Loss Behaviours and Sleep

Model fit for all specified models was very good (Table 4). Weight loss behaviours did not significantly predict experiencing sleep difficulties overall; however, a significant longitudinal association was identified for ‘falling asleep at inappropriate times’ (*β* = 0.16, SE = 0.04, *p* < 0.001) and for shorter sleep duration (*β* = 0.06, SE = 0.02, *p* < 0.01).

**TABLE 4:** Longitudinal regression analyses predicting sleep difficulties and behaviours.

Model	Predictor	Beta	SE	*p*	*p* (adjusted)	Fit indices
Model 1—Sleep difficulties	Restrictive eating	0.05	0.02	0.07	0.14	*χ* ^2^ (8, 5705) = 161.77, CFI = 0.98, TLI = 0.98, RMSEA = 0.06, SRMR = 0.02
	**Gender**	−0.13***	**0.04**	***p* < 0.001**	***p* < 0.001**	
	BMI—cat	−0.01	0.04	0.61	0.94	
	Household income	0.02	0.01	0.28	0.42	
Model 2—Sleep onset difficulties	Restrictive eating	0.04	0.03	0.11	0.14	*χ* ^2^ (8, 5705) = 162.82, CFI = 0.97, TLI = 0.98, RMSEA = 0.06, SRMR = 0.02
	**Gender**	−0.10***	**0.03**	***p* < 0.001**	***p* < 0.001**	
	BMI—cat	−0.002	0.04	0.94	0.94	
	Household income	−0.003	0.01	0.89	0.89	
Model 3—Wake after sleep onset	Restrictive eating	0.02	0.03	0.55	0.62	*χ* ^2^ (8, 5705) = 164.63, CFI = 0.97, TLI = 0.98, RMSEA = 0.06, SRMR = 0.02
	**Gender**	−0.21***	**0.05**	***p* < 0.001**	***p* < 0.001**	
	**BMI—cat**	−0.06*	**0.05**	**0.01**	0.09	
	Household income	0.03	0.02	0.18	0.41	
Model 4—Early awakening	Restrictive eating	−0.005	0.04	0.91	0.91	*χ* ^2^ (8, 5705) = 158.66, CFI = 0.98, TLI = 0.98, RMSEA = 0.06, SRMR = 0.02
	**Gender**	−0.07*	**0.06**	**0.04**	0.05	
	BMI—cat	−0.02	0.07	0.64	0.94	
	Household income	−0.04	0.02	0.23	0.41	
Model 5—Difficulties with waking up	Restrictive eating	0.06	0.03	0.07	0.14	*χ*2 (8, 5705) = 165.14, CFI = 0.97, TLI = 0.98, RMSEA = 0.06, SRMR = 0.02
	**Gender**	−0.12***	**0.05**	***p* < 0.001**	***p* < 0.001**	
	BMI—cat	−0.003	0.05	0.90	0.94	
	Household income	0.005	0.02	0.82	0.89	
Model 6—Sleep disruptions	Restrictive eating	0.09	0.05	0.09	0.14	*χ*2 (8, 5705) = 162.89, CFI = 0.97, TLI = 0.98, RMSEA = 0.06, SRMR = 0.02
	Gender	−0.03	0.07	0.39	0.39	
	BMI—cat	0.05	0.08	0.26	0.78	
	Household income	0.06	0.03	0.10	0.30	
Model 7—Falling asleep at inappropriate times	**Restrictive eating**	0.16***	**0.04**	***p* < 0.001**	***p* < 0.001**	*χ* 2 (8, 5705) = 159.90, CFI = 0.98, TLI = 0.98, RMSEA = 0.06, SRMR = 0.02
	**Gender**	−0.06*	**0.06**	**0.03**	**0.045**	
	BMI—cat	0.03	0.06	0.42	0.94	
	Household income	0.05	0.02	0.08	0.30	
Model 8—Time‐in‐bed	Restrictive eating	0.03	0.02	0.11	0.14	*χ*2 (8, 5705) = 160.19, CFI = 0.98, TLI = 0.98, RMSEA = 0.06, SRMR = 0.01
	**Gender**	0.09***	**0.03**	***p* < 0.001**	***p* < 0.001**	
	BMI—cat	−0.003	0.03	0.86	0.94	
	**Household income**	−0.07***	**0.01**	***p* < 0.001**	***p* < 0.001**	
Model 9—Sleep duration	**Restrictive eating**	0.06**	**0.02**	**0.001**	**0.005**	*χ*2 (8, 5705) = 168.50, CFI = 0.96, TLI = 0.92, RMSEA = 0.06, SRMR = 0.01
	Gender	−0.03	0.03	0.06	0.09	
	BMI—cat	−0.02	0.03	0.20	0.36	
	Household income	0.008	0.01	0.56	0.78	

*Note:* ***p < 0.001, **p < 0.01, *p < 0.05; for restrictive eating, lower values represent more restrictive eating behaviours; equally for all individual sleep problems, lower values represent presence of sleep problems. Bold values indicate significant associations.

### 3.3 Mediation

**FIGURE 1:**
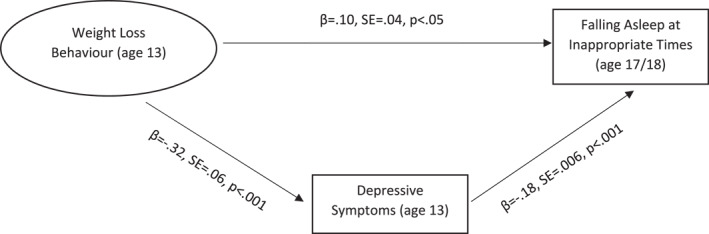
Mediation model illustrating the association between weight loss behaviour and falling asleep at inappropriate times as mediated by depressive symptoms. Indirect effect: *β* = 0.06, SE = 0.008, *p* < 0.001. Total effect: *β* = 0.16, SE = 0.04, *p* < 0.001. Model fit: *χ* ^2^ (10, 5705) = 164.51, CFI = 0.98, TLI = 0.98, RMSEA = 0.05, SRMR = 0.01.

**FIGURE 2:**
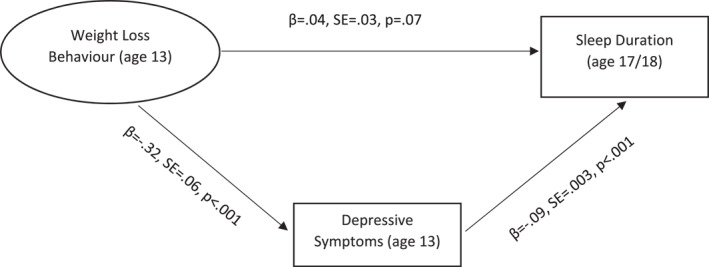
Mediation model illustrating the association between weight loss behaviour and self‐reported sleep duration as mediated by depressive symptoms. Indirect effect: *β* = 0.03, SE = 0.004, *p* < 0.001. Total effect: *β* = 0.10, SE = 0.02, *p* < 0.01. Model fit: *χ* ^2^ (10, 5705) = 169.16, CFI = 0.98, TLI = 0.98, RMSEA = 0.06, SRMR = 0.01.

Mediation models were tested for all significant regression models, specifying depressive symptoms as mediator (Figures 1 and 2). Significant mediation effects were found for both mediation models. Depressive symptoms partially mediated the association between weight loss behaviours and subsequent problems of falling asleep at inappropriate times. The association between weight loss behaviours and subsequent shorter sleep duration, in contrast, was fully mediated by depressive symptoms.

### 3.4 Moderation

A multi‐group SEM was conducted to examine whether excessive online behaviours moderated the relationship between weight loss behaviours and sleep. All specified models demonstrated a good fit. However, none of the tested paths from weight loss behaviours to sleep behaviours and problems differed significantly across groups, including both week‐ and weekend behaviours (see Supporting Information S2).

### 3.5 Explorative Post‐Hoc Analyses

Considering that recommendations for inappropriate sleep times include both too few and too many hours spent sleeping, we conducted additional exploratory post‐hoc analyses to investigate the effects of both too short (< 8 h) and excessively long (> 10 h) sleep durations and times‐in‐bed (Paruthi et al. 2016). As outlined in the Supporting Information S3, only *short* sleep duration (OR = 0.94, 95% CI [0.90, 0.98], *p* < 0.01) and times‐in‐bed (OR = 0.94, 95% CI [0.89, 0.99], *p* < 0.05) < 8 h were significantly predicted by weight loss behaviours.

## 4 Discussion

In the present national Irish cohort, 13‐year‐olds’ weight loss behaviours significantly predicted adolescents' shorter sleep duration and short time‐spent‐in‐bed (< 8 h) four to five years later. This longitudinal finding is in line with clinical research, reporting shorter sleep times in patients with restrictive‐type anorexia nervosa (Romigi et al. 2022). However, evidence in this area is mixed (Abanobi et al. 2025) and requires further investigation. Shorter sleep periods might reflect a desire to maximise times of energy expenditure as well as the impacts of sustained fasting on sleep regulation, which have equally been identified in animal research (Alvarenga et al. 2005). Importantly, in the present non‐clinical population, the association between weight loss behaviours and shorter sleep was fully mediated by depressive symptoms. These findings replicate and extend findings from a previous project in a British cohort (Opitz et al. 2025), which identified depressive symptoms as a putative mediator for the relationship between weight loss behaviours and subsequent poorer sleep in British adolescents. Consequently, findings from both studies indicate mood as a promising target to limit or prevent poorer and insufficient sleep as a consequence of weight loss behaviours. Interestingly, rates of weight loss behaviours and rates of sleep difficulties assessed across both samples, were slightly lower in Irish adolescents compared to the one‐year older British participants, which could be explained through age‐, as well as cultural differences. Further cross‐cultural comparisons could shed further light on these differences.

One limitation of this and previous work (Opitz et al. 2025), is that the GUI does not include repeated assessments of constructs, with depressive symptoms being reported at the same timepoint as weight loss behaviours (age 13). Thus, temporal and causal associations require further longitudinal investigation. Moreover, the current study could not account for bioregulatory changes in developmental sleep trajectories across adolescence (Carskadon 2011). That said, the GUI allows for the differentiation between time‐in‐bed and sleep duration, which were both predicted by weight loss behaviours when investigating time durations below 8 hours.

Excessive online behaviours, as measured in the GUI, did not moderate the relationship between weight loss behaviours and sleep. However, time‐spent‐online may not accurately depict *problematic* online engagement, as it doesn't provide sufficient insights into the addictive quality of online behaviour. Further research is needed to explore the addictive nature and timing of online behaviour, as well as the role of specific online behaviours (e.g., social media engagement, watching series/movies, messaging) to disentangle the effects of screen time (Chan et al. 2021) from content exposure (McNicholas et al. 2009) on young people's sleep and eating behaviours.

Interestingly, the current study found a significant relationship between weight loss behaviours and problems with falling asleep at inappropriate times, that was only partially mediated through depressive symptoms. While previous research has found associations between young people's dieting behaviours and their sleep‐related daytime dysfunction, potentially reflecting adolescents' increased daytime sleepiness (Hirai et al. 2022), research in this area is scarce. Depending on how young people interpreted ‘inappropriate times’, our finding could reflect participants' exhaustion/sleepiness, as well as a misalignment of sleep schedules. Future research will need to investigate how weight loss behaviours could be associated with different sleep timings as well as general exhaustion.

Overall, this study provides additional support of the potential role of depressive symptoms in the relationship between weight loss behaviours and sleep.

## Author Contributions

**Marie‐Christine Opitz:** conceptualization, methodology, formal analysis, investigation, data curation, writing – original draft, project administration, writing – review and editing. **Sarah Cooney:** methodology, supervision, writing – review and editing. **Nora Trompeter:** methodology, writing – review and editing. **Sylvane Desrivières:** methodology, writing – review and editing, funding acquisition. **Nadia Micali:** methodology, writing – review and editing, funding acquisition. **Ulrike Schmidt:** conceptualization, writing – review and editing, funding acquisition. **Helen Sharpe:** conceptualization, methodology, supervision, writing – review and editing, funding acquisition.

## Funding

The research leading to these results has received funding from the European Union's Horizon 2020 research and innovation programme under grant agreement No. 101008589, COORDINATE—COhort cOmmunity Research and Development Infrastructure Network for Access Throughout Europe. This work was further supported by the Medical Research Council/Arts and Humanities Research Council/Economic and Social Research Council Adolescence, Mental Health and the Developing Mind initiative as part of the EDIFY programme (Grant MR/W002418/1). HS is supported by UK Research and Innovation (MRC, ESRC, AHRC), the National Institute for Health and Care Research and the Medical Research Foundation as part of the EDAC network (Grant MR/X03058X/1). US receives salary support from the National Institute of Health Research (NIHR) Biomedical Research Centre for Mental Health, South London and Maudsley NHS Foundation Trust and Institute of Psychiatry, Psychology and Neuroscience, King's College London. The views expressed in this publication are those of the authors and not necessarily those of the National Health Service, the NIHR or the UK Department of Health.

## Ethics Statement

Ethical approval was sought and granted by the University of Edinburgh Research Ethics Committee (Reference: 22‐23CLPS066).

## Conflicts of Interest

Nora Trompeter receives an honorarium from Wiley as Associate Editor for Mental Health Science. Nadia Micali receives an honorarium as associate editor on European Eating Disorders review.

## Supporting information


Supporting Information S1

Supporting Information S2

Supporting Information S3

## Data Availability

This study's protocol was registered on OSF (https://doi.org/10.17605/OSF.IO/G2PTM). Growing Up in Ireland datasets are made available to researchers on a confidential and anonymised basis through the Irish Social Sciences Data Archive. These files are known as Anonymised Microdata Files (AMFs).
