# Supplementary material for: Investigating the Prospective Relationship Between Weight Loss Behaviours and Sleep in Adolescents From the Growing Up in Ireland Cohort
Source: Eur Eat Disord Rev. 2025 Oct 25;34(2):455–63. doi: 10.1002/erv.70045 (PMC12862554; doi:10.1002/erv.70045)
Supplement: Supplementary file 1 — Supporting Information S1 [file ERV-34-455-s002.docx]

**Supplementary Material 1**

Supplementary Table 1. Correlations Between Weight Loss Behaviour Variables, General Sleep Difficulties, Depressive Symptoms, and Online Behaviour

|  | Weight Loss Intention (age 13) | Dietary Restriction (age 13) | Excessive Exercise (age 13) | Self-Weighing (age 13) | Sleep Difficulties (age 17/18) | Depressive Symptoms (age 13) | Online Behaviour Week (age 17/18) | Online Behaviour Weekend (age 17/18) |
| --- | --- | --- | --- | --- | --- | --- | --- | --- |
| Weight Loss Intention (age 13) | 1 |  |  |  |  |  |  |  |
| Dietary Restriction (age 13) | .51***  [.49; .53] | 1 |  |  |  |  |  |  |
| Excessive Exercise (age 13) | .49***  [.47; .51] | .53***  [.51; .55] | 1 |  |  |  |  |  |
| Self-Weighing (age 13) | .13***  [.10; .16] | .13***  [.10; .16] | .09***  [.06; .11] | 1 |  |  |  |  |
| Sleep Difficulties (age 17) | .04**†  [.02; .07] | .04**  [.02; .07] | .03†  [.001; .05] | .02  [-.004; .05] | 1 |  |  |  |
| Depressive Symptoms (age 13) | -.17***  [-.19; -.14] | -.21***  [-.23; -.18] | -.15***  [-.18; -.13] | -.10***  [-.13; -.08] | -.17***  [-.19; -.14] | 1 |  |  |
| Online Behaviour Week (age 17/18) | .09***  [.06; .11] | .05***  [.03; .08] | .05***  [.02; .08] | .002  [-.02; .03] | .07***  [.04; .09] | -.08***  [-.10; -.05] | 1 |  |
| Online Behaviour Weekend (age 17/18) | .04*†  [.01; .06] | .02  [-.006; .05] | .01  [-.02; .04] | - .005  [-.03; .02] | .05***  [.02; .08] | -.03†  [-.05; -.001] | .49***  [.47; .51] | 1 |
| *p<.05, **p<.01, ***p<.001; correlations between dichotomous variables are based on phi coefficients; correlations with depressive symptoms were based on point-biserial correlation coefficients; significance testing was conducted using chi-square tests, p-values were adjusted for multiple testing using the Benjamini-Hochberg correction (changes indicated by †); lower values on weight loss behaviours, sleep difficulties, and online behaviours indicate affirmative answers; for depressive symptoms, higher values indicate more depressive symptoms | | | | | | | | |

Supplementary Table 2. Correlations Between Individual Sleep Difficulties and All Other Variables:

|  | Weight Loss Intention (age 13) | Dietary Restriction (age 13) | Excessive Exercise (age 13) | Self-Weighing (age 13) | Depressive Symptoms (age 13) | Online Behaviour Week (age 17/18) | Online Behaviour Weekend (age 17/18) |
| --- | --- | --- | --- | --- | --- | --- | --- |
| Sleep Onset Difficulty (age 17/18) | .04*†  [.01; .06] | .03*  [.007; .06] | .02  [-.007; .05] | .01  [-.01; .04] | -.13***  [-.15; -.10] | .05***  [.03; .08] | .05***  [.03; .08] |
| Wake After Sleep Onset (age 17/18) | .04**  [.01; .06] | .04**  [.01; .07] | .02  [-.01; .04] | .03  [.001; .05] | -.15***  [-.17; -.12] | .07***  [.05; .10] | .05***  [.03; .08] |
| Early Awakening (age 17/18) | .007  [-.02; .03] | .02  [-.01; .04] | .004  [-.02; .03] | .03  [.001; .05] | -.06***  [-.09; -.04] | .01  [-.01; .04] | .006  [-.02; .03] |
| Difficulty With Waking (age 17/18) | .05***  [.02; .07] | .03†  [.001; .05] | .03  [-.001; .05] | .002  [-.03; .02] | -.11***  [-.14; -.09] | .08***  [.05; .11] | .06***  [.03; .09] |
| Sleep Disruption (age 17/18) | .03  [-.001; .05] | .02  [-.009; .04] | .01  [-.02; .04] | .008  [-.02; .03] | -.07***  [-.10; -.05] | .03  [-.001; .05] | .02  [-.01; .04] |
| Falling Asleep at Inappropriate Times (age 17/18) | .05***  [.03; .09] | .05***  [.02; .08] | .05***  [.03; .08] | .01  [-.01; .04] | -.12***  [-.15; -.10] | .08***  [.05; .10] | .05***  [.02; .08] |

*p<.05, **p<.01, ***p<.001; correlations between dichotomous variables are based on phi coefficients; correlations with depressive symptoms were based on point-biserial correlation coefficients; significance testing was conducted using chi-square tests, p-values were adjusted for multiple testing using the Benjamini-Hochberg correction (changes indicated by †); lower values on weight loss behaviours, sleep difficulties, and online behaviours indicate affirmative answers; for depressive symptoms, higher values indicate more depressive symptoms
