# Supplementary material for: Investigating the Prospective Relationship Between Weight Loss Behaviours and Sleep in Adolescents From the Growing Up in Ireland Cohort
Source: Eur Eat Disord Rev. 2025 Oct 25;34(2):455–63. doi: 10.1002/erv.70045 (PMC12862554; doi:10.1002/erv.70045)
Supplement: Supplementary file 2 — Supporting Information S2 [file ERV-34-455-s001.docx]

**Supplementary Material 2**

Supplementary Table 3. Moderation of Excessive Online Behaviour – Week Behaviours

| Sleep Difficulties | | | | | | |
| --- | --- | --- | --- | --- | --- | --- |
| Model | Predictor | Beta | SE | p | P (adjusted) | Fit Indices |
| Model 1 – Sleep difficulties  Group 1 (online < 3h a day) | Weight Loss Behaviour | 0.02 | 0.03 | 0.42 |  | χ^2^(16, 4453)= 122.05, CFI=.98, TLI=.98, RMSEA=.06, SRMR=.02 |
|  | **Gender** | **-0.13** | **0.04** | **<.001** |  |  |
|  | BMI – cat | -0.03 | 0.05 | 0.30 |  |  |
|  | Household Income | 0.02 | 0.02 | 0.42 |  |  |
| Model 2 – Sleep difficulties  Group 2 (online > 3h a day) | **Weight Loss Behaviour** | **0.11** | **0.05** | **<.05** |  | χ^2^(16, 1198)= 39.27, CFI=.98, TLI=.98, RMSEA=.06, SRMR=.02 |
|  | **Gender** | **-0.12** | **0.08** | **<.01** |  |  |
|  | BMI – cat | 0.04 | 0.08 | 0.34 |  |  |
|  | Household Income | 0.02 | 0.03 | 0.53 |  |  |
| Sleep Onset Difficulties | | | | | | |
| Model | Predictor | Beta | SE | p | P (adjusted) | Fit Indices |
| Model 1 – Sleep Onset difficulties  Group 1 (online < 3h a day) | Weight Loss Behaviour | 0.03 | 0.03 | 0.42 |  | χ^2^(16, 4453)= 122.80, CFI=.98, TLI=.98, RMSEA=.06, SRMR=.02 |
|  | **Gender** | **-0.10** | **0.05** | **<.001** |  |  |
|  | BMI – cat | -0.01 | 0.05 | 0.77 |  |  |
|  | Household Income | -0.01 | 0.02 | 0.72 |  |  |
| Model 2 – Sleep Onset difficulties  Group 2 (online > 3h a day) | Weight Loss Behaviour | 0.09 | 0.05 | 0.12 |  | χ^2^(16, 1198)= 37.19, CFI=.98, TLI=.98, RMSEA=.06, SRMR=.02 |
|  | Gender | -0.08 | 0.08 | 0.07 |  |  |
|  | BMI – cat | 0.03 | 0.08 | 0.51 |  |  |
|  | Household Income | 0.01 | 0.03 | 0.86 |  |  |
| Wake After Sleep Onset | | | | | | |

| Model | Predictor | Beta | SE | p | P (adjusted) | Fit Indices |
| --- | --- | --- | --- | --- | --- | --- |
| Model 1 – WASO  Group 1 (online < 3h a day) | Weight Loss Behaviour | -0.02 | 0.03 | 0.56 |  | χ^2^(16, 4453)= 123.92, CFI=.98, TLI=.98, RMSEA=.06, SRMR=.02 |
|  | **Gender** | **-0.18** | **0.05** | **<.001** |  |  |
|  | BMI – cat | -0.08 | 0.05 | <.01 |  |  |
|  | Household Income | 0.04 | 0.02 | 0.17 |  |  |
| Model 2 – WASO  Group 2 (online > 3h a day) | Weight Loss Behaviour | 0.10 | 0.06 | 0.12 |  | χ^2^(16, 1198)= 39.27, CFI=.98, TLI=.98, RMSEA=.06, SRMR=.02 |
|  | **Gender** | **-0.27** | **0.10** | **<.001** |  |  |
|  | BMI – cat | -0.01 | 0.09 | 0.82 |  |  |
|  | Household Income | 0.003 | 0.03 | 0.95 |  |  |
| Early Wakeup | | | | | | |
| Model | Predictor | Beta | SE | p | P (adjusted) | Fit Indices |
| Model 1 – Early Wakeup  Group 1 (online < 3h a day) | Weight Loss Behaviour | 0.01 | 0.04 | 0.82 |  | χ^2^(16, 4453)= 120.72, CFI=.98, TLI=.98, RMSEA=.06, SRMR=.02 |
|  | Gender | -0.05 | 0.07 | 0.16 |  |  |
|  | BMI – cat | -0.04 | 0.08 | 0.35 |  |  |
|  | Household Income | -0.06 | 0.03 | 0.09 |  |  |
| Model 2 – Early Wakeup  Group 2 (online > 3h a day) | Weight Loss Behaviour | -0.03 | 0.08 | 0.75 |  | χ^2^(16, 1198)= 36.66, CFI=.98, TLI=.98, RMSEA=.06, SRMR=.02 |
|  | Gender | -0.11 | 0.15 | 0.14 |  |  |
|  | BMI – cat | 0.09 | 0.17 | 0.38 |  |  |
|  | Household Income | 0.02 | 0.05 | 0.75 |  |  |
| Difficulty Waking Up | | | | | | |
| Model | Predictor | Beta | SE | p | P (adjusted) | Fit Indices |
| Model 1 – Difficulty Waking Up  Group 1 (online < 3h a day) | Weight Loss Behaviour | 0.04 | 0.03 | 0.35 |  | χ^2^(16, 4453)= 125.04, CFI=.98, TLI=.98, RMSEA=.06, SRMR=.02 |
|  | **Gender** | **-0.13** | **0.05** | **<.001** |  |  |
|  | BMI – cat | -0.02 | 0.06 | 0.43 |  |  |
|  | Household Income | 0.01 | 0.02 | 0.64 |  |  |
| Model 2 – Difficulty Waking Up  Group 2 (online > 3h a day) | Weight Loss Behaviour | 0.08 | 0.06 | 0.19 |  | χ^2^(16, 1198)= 36.69, CFI=.98, TLI=.98, RMSEA=.06, SRMR=.02 |
|  | Gender | -0.07 | 0.09 | 0.11 |  |  |
|  | BMI – cat | 0.07 | 0.09 | 0.22 |  |  |
|  | Household Income | -0.04 | 0.03 | 0.44 |  |  |
| Sleep Disruption | | | | | | |
| Model | Predictor | Beta | SE | p | P (adjusted) | Fit Indices |
| Model 1 – Sleep Disruption  Group 1 (online < 3h a day) | Weight Loss Behaviour | 0.07 | 0.05 | 0.25 |  | χ^2^(16, 4453)= 124.40, CFI=.98, TLI=.98, RMSEA=.06, SRMR=.02 |
|  | Gender | -0.04 | 0.09 | 0.38 |  |  |
|  | BMI – cat | 0.03 | 0.09 | 0.55 |  |  |
|  | Household Income | 0.07 | 0.03 | 0.07 |  |  |
| Model 2 – Sleep Disruption  Group 2 (online > 3h a day) | Weight Loss Behaviour | 0.11 | 0.09 | 0.25 |  | χ^2^(16, 1198)= 36.97, CFI=.98, TLI=.98, RMSEA=.06, SRMR=.02 |
|  | Gender | -0.02 | 0.15 | 0.80 |  |  |
|  | BMI – cat | 0.14 | 0.16 | 0.14 |  |  |
|  | Household Income | 0.02 | 0.06 | 0.84 |  |  |
| Sleepiness | | | | | | |
| Model | Predictor | Beta | SE | p | P (adjusted) | Fit Indices |
| Model 1 – Sleepiness  Group 1 (online < 3h a day) | **Weight Loss Behaviour** | **0.17** | **0.04** | **<.001** |  | χ^2^(16, 4453)= 119.87, CFI=.98, TLI=.98, RMSEA=.06, SRMR=.02 |
|  | Gender | -0.05 | 0.07 | 0.17 |  |  |
|  | BMI – cat | 0.04 | 0.07 | 0.25 |  |  |
|  | Household Income | 0.07 | 0.03 | 0.05 |  |  |
| Model 2 – Sleepiness  Group 2 (online > 3h a day) | Weight Loss Behaviour | 0.13 | 0.06 | 0.06 |  | χ^2^(16, 1198)= 36.07, CFI=.98, TLI=.98, RMSEA=.06, SRMR=.02 |
|  | Gender | -0.09 | 0.12 | 0.13 |  |  |
|  | BMI – cat | 0.02 | 0.10 | 0.73 |  |  |
|  | Household Income | -0.01 | 0.04 | 0.85 |  |  |
| Time in Bed | | | | | | |
| Model | Predictor | Beta | SE | p | P (adjusted) | Fit Indices |
| Model 1 – Time in Bed  Group 1 (online < 3h a day) | Weight Loss Behaviour | 0.03 | 0.02 | 0.21 |  | χ^2^(16, 4453)= 121.78, CFI=.98, TLI=.98, RMSEA=.06, SRMR=.02 |
|  | **Gender** | **0.07** | **0.04** | **<.001** |  |  |
|  | BMI – cat | -0.02 | 0.04 | 0.35 |  |  |
|  | **Household Income** | **-0.07** | **0.01** | **<.001** |  |  |
| Model 2 – Time in Bed  Group 2 (online > 3h a day) | Weight Loss Behaviour | 0.04 | 0.04 | 0.44 |  | χ^2^(16, 1198)= 42.50, CFI=.98, TLI=.98, RMSEA=.06, SRMR=.02 |
|  | **Gender** | **0.15** | **0.07** | **<.001** |  |  |
|  | BMI – cat | 0.03 | 0.07 | 0.43 |  |  |
|  | **Household Income** | **-0.08** | **0.02** | **<.05** |  |  |
| Sleep Duration | | | | | | |
| Model | Predictor | Beta | SE | p | P (adjusted) | Fit Indices |
| Model 1 – Sleep Duration  Group 1 (online < 3h a day) | **Weight Loss Behaviour** | **0.05** | **0.02** | **<.05** |  | χ^2^(16, 4453)= 122.11, CFI=.98, TLI=.98, RMSEA=.06, SRMR=.02 |
|  | **Gender** | **-0.03** | **0.03** | **<.05** |  |  |
|  | BMI – cat | -0.03 | 0.03 | 0.07 |  |  |
|  | Household Income | 0.004 | 0.01 | 0.80 |  |  |
| Model 2 – Sleep Duration  Group 2 (online > 3h a day) | **Weight Loss Behaviour** | **0.10** | **0.04** | **<.05** |  | χ^2^(16, 1198)= 39.24, CFI=.98, TLI=.98, RMSEA=.06, SRMR=.02 |
|  | Gender | 0.03 | 0.07 | 0.37 |  |  |
|  | BMI – cat | 0.03 | 0.06 | 0.46 |  |  |
|  | Household Income | -0.02 | 0.02 | 0.49 |  |  |

Supplementary Table 4. Moderation of Excessive Online Behaviour – Weekend Behaviours

| Sleep Difficulties | | | | | | |
| --- | --- | --- | --- | --- | --- | --- |
| Model | Predictor | Beta | SE | p | P (adjusted) | Fit Indices |
| Model 1 – Sleep difficulties  Group 1 (online < 3h a day) | Weight Loss Behaviour | 0.04 | 0.03 | 0.28 |  | χ^2^(16, 3512)= 94.31, CFI=.98, TLI=.98, RMSEA=.06, SRMR=.02 |
|  | **Gender** | **-0.16** | **0.05** | **<.001** |  |  |
|  | BMI – cat | -0.003 | 0.05 | 0.91 |  |  |
|  | Household Income | 0.03 | 0.02 | 0.20 |  |  |
| Model 2 – Sleep difficulties  Group 2 (online > 3h a day) | Weight Loss Behaviour | 0.06 | 0.04 | 0.12 |  | χ^2^(16, 2139)= 68.81, CFI=.98, TLI=.98, RMSEA=.06, SRMR=.02 |
|  | **Gender** | **-0.07** | **0.06** | **<.05** |  |  |
|  | BMI – cat | -0.02 | 0.06 | 0.57 |  |  |
|  | Household Income | 0.01 | 0.02 | 0.72 |  |  |
| Sleep Onset Difficulties | | | | | | |
| Model | Predictor | Beta | SE | p | P (adjusted) | Fit Indices |
| Model 1 – Sleep Onset difficulties  Group 1 (online < 3h a day) | Weight Loss Behaviour | 0.03 | 0.03 | 0.46 |  | χ^2^(16, 3512)= 95.26, CFI=.98, TLI=.98, RMSEA=.06, SRMR=.02 |
|  | **Gender** | **-0.12** | **0.05** | **<.001** |  |  |
|  | BMI – cat | 0.02 | 0.06 | 0.50 |  |  |
|  | Household Income | 0.001 | 0.02 | 0.98 |  |  |
| Model 2 – Sleep Onset difficulties  Group 2 (online > 3h a day) | Weight Loss Behaviour | 0.07 | 0.04 | 0.12 |  | χ^2^(16, 2139)= 69.14, CFI=.98, TLI=.98, RMSEA=.06, SRMR=.02 |
|  | Gender | -0.05 | 0.06 | 0.13 |  |  |
|  | BMI – cat | -0.02 | 0.06 | 0.48 |  |  |
|  | Household Income | -0.002 | 0.02 | 0.95 |  |  |
| Wake After Sleep Onset | | | | | | |
| Model | Predictor | Beta | SE | p | P (adjusted) | Fit Indices |
| Model 1 – WASO  Group 1 (online < 3h a day) | Weight Loss Behaviour | 0.03 | 0.04 | 0.56 |  | χ^2^(16, 3512)= 98.84, CFI=.97, TLI=.98, RMSEA=.06, SRMR=.02 |
|  | **Gender** | **-0.20** | **0.06** | **<.001** |  |  |
|  | BMI – cat | -0.08 | 0.06 | 0.02 |  |  |
|  | Household Income | 0.05 | 0.02 | 0.011 |  |  |
| Model 2 – WASO  Group 2 (online > 3h a day) | Weight Loss Behaviour | 0.003 | 0.05 | 0.95 |  | χ^2^(16, 2139)= 70.09, CFI=.97, TLI=.98, RMSEA=.06, SRMR=.02 |
|  | **Gender** | **-0.21** | **0.07** | **<.001** |  |  |
|  | BMI – cat | -0.05 | 0.07 | 0.23 |  |  |
|  | Household Income | 0.009 | 0.03 | 0.79 |  |  |
| Early Wakeup | | | | | | |
| Model | Predictor | Beta | SE | p | P (adjusted) | Fit Indices |
| Model 1 – Early Wakeup  Group 1 (online < 3h a day) | Weight Loss Behaviour | 0.04 | 0.05 | 0.45 |  | χ^2^(16, 3512)= 92.66, CFI=.98, TLI=.98, RMSEA=.06, SRMR=.02 |
|  | **Gender** | **-0.08** | **0.08** | **<.05** |  |  |
|  | BMI – cat | -0.01 | 0.09 | 0.79 |  |  |
|  | Household Income | -0.03 | 0.03 | 0.37 |  |  |
| Model 2 – Early Wakeup  Group 2 (online > 3h a day) | Weight Loss Behaviour | -0.07 | 0.06 | 0.35 |  | χ^2^(16, 2139)= 70.53, CFI=.98, TLI=.98, RMSEA=.06, SRMR=.02 |
|  | Gender | -0.03 | 0.11 | 0.54 |  |  |
|  | BMI – cat | -0.01 | 0.11 | 0.92 |  |  |
|  | Household Income | -0.05 | 0.04 | 0.39 |  |  |
| Difficulty Waking Up | | | | | | |
| Model | Predictor | Beta | SE | p | P (adjusted) | Fit Indices |
| Model 1 – Difficulty Waking Up  Group 1 (online < 3h a day) | Weight Loss Behaviour | 0.02 | 0.04 | 0.64 |  | χ^2^(16, 3512)= 96.14, CFI=.97, TLI=.98, RMSEA=.06, SRMR=.02 |
|  | **Gender** | **-0.14** | **0.06** | **<.001** |  |  |
|  | BMI – cat | -0.01 | 0.06 | 0.67 |  |  |
|  | Household Income | 0.03 | 0.02 | 0.35 |  |  |
| Model 2 – Difficulty Waking Up  Group 2 (online > 3h a day) | **Weight Loss Behaviour** | **0.09** | **0.04** | **<.05** |  | χ^2^(16, 2139)= 71.53, CFI=.97, TLI=.98, RMSEA=.06, SRMR=.02 |
|  | **Gender** | **-0.08** | **0.07** | **<.05** |  |  |
|  | BMI – cat | 0.02 | 0.07 | 0.63 |  |  |
|  | Household Income | -0.02 | 0.03 | 0.48 |  |  |
| Sleep Disruption | | | | | | |
| Model | Predictor | Beta | SE | p | P (adjusted) | Fit Indices |
| Model 1 – Sleep Disruption  Group 1 (online < 3h a day) | Weight Loss Behaviour | 0.06 | 0.06 | 0.36 |  | χ^2^(16, 3512)= 98.62, CFI=.97, TLI=.98, RMSEA=.06, SRMR=.03 |
|  | Gender | -0.06 | 0.10 | 0.18 |  |  |
|  | BMI – cat | 0.04 | 0.10 | 0.48 |  |  |
|  | **Household Income** | **0.15** | **0.03** | **<.01** |  |  |
| Model 2 – Sleep Disruption  Group 2 (online > 3h a day) | Weight Loss Behaviour | 0.13 | 0.08 | 0.13 |  | χ^2^(16, 2139)= 70.07, CFI=.97, TLI=.98, RMSEA=.06, SRMR=.03 |
|  | Gender | 0.01 | 0.12 | 0.93 |  |  |
|  | BMI – cat | 0.09 | 0.14 | 0.23 |  |  |
|  | Household Income | -0.08 | 0.05 | 0.23 |  |  |
| Sleepiness | | | | | | |
| Model | Predictor | Beta | SE | p | P (adjusted) | Fit Indices |
| Model 1 – Sleepiness  Group 1 (online < 3h a day) | **Weight Loss Behaviour** | **0.18** | **0.05** | **<.01** |  | χ^2^(16, 3512)= 92.66, CFI=.98, TLI=.98, RMSEA=.06, SRMR=.02 |
|  | Gender | -0.08 | 0.08 | 0.06 |  |  |
|  | BMI – cat | 0.04 | 0.08 | 0.34 |  |  |
|  | **Household Income** | **0.11** | **0.03** | **<.01** |  |  |
| Model 2 – Sleepiness  Group 2 (online > 3h a day) | **Weight Loss Behaviour** | **0.15** | **0.05** | **<.01** |  | χ^2^(16, 2139)= 68.28, CFI=.98, TLI=.98, RMSEA=.06, SRMR=.02 |
|  | Gender | -0.05 | 0.09 | 0.31 |  |  |
|  | BMI – cat | 0.03 | 0.09 | 0.49 |  |  |
|  | Household Income | -0.02 | 0.03 | 0.69 |  |  |
| Time in Bed | | | | | | |
| Model | Predictor | Beta | SE | p | P (adjusted) | Fit Indices |
| Model 1 – Time in Bed  Group 1 (online < 3h a day) | Weight Loss Behaviour | 0.02 | 0.02 | 0.54 |  | χ^2^(16, 3512)= 93.13, CFI=.98, TLI=.98, RMSEA=.06, SRMR=.02 |
|  | **Gender** | **0.06** | **0.04** | **<.01** |  |  |
|  | BMI – cat | -0.01 | 0.04 | 0.78 |  |  |
|  | **Household Income** | **-0.09** | **0.01** | **<.001** |  |  |
| Model 2 – Time in Bed  Group 2 (online > 3h a day) | Weight Loss Behaviour | 0.06 | 0.03 | 0.08 |  | χ^2^(16, 2139)= 68.62, CFI=.98, TLI=.98, RMSEA=.06, SRMR=.02 |
|  | **Gender** | **0.13** | **0.05** | **<.001** |  |  |
|  | BMI – cat | -0.01 | 0.05 | 0.78 |  |  |
|  | Household Income | -0.04 | 0.02 | 0.13 |  |  |
| Sleep Duration | | | | | | |
| Model | Predictor | Beta | SE | p | P (adjusted) | Fit Indices |
| Model 1 – Sleep Duration  Group 1 (online < 3h a day) | **Weight Loss Behaviour** | **0.06** | **0.02** | **<.05** |  | χ^2^(16, 3512)= 95.20, CFI=.98, TLI=.98, RMSEA=.06, SRMR=.02 |
|  | **Gender** | **-0.05** | **0.04** | **<.05** |  |  |
|  | BMI – cat | -0.02 | 0.04 | 0.37 |  |  |
|  | Household Income | -0.01 | 0.01 | 0.61 |  |  |
| Model 2 – Sleep Duration  Group 2 (online > 3h a day) | **Weight Loss Behaviour** | **0.08** | **0.03** | **<.05** |  | χ^2^(16, 2139)= 70.09, CFI=.98, TLI=.98, RMSEA=.06, SRMR=.02 |
|  | Gender | 0.03 | 0.05 | 0.24 |  |  |
|  | BMI – cat | -0.02 | 0.05 | 0.46 |  |  |
|  | Household Income | 0.02 | 0.02 | 0.35 |  |  |
