# Supplementary material for: Investigating the Prospective Relationship Between Weight Loss Behaviours and Sleep in Adolescents From the Growing Up in Ireland Cohort
Source: Eur Eat Disord Rev. 2025 Oct 25;34(2):455–63. doi: 10.1002/erv.70045 (PMC12862554; doi:10.1002/erv.70045)
Supplement: Supplementary file 3 — Supporting Information S3 [file ERV-34-455-s003.docx]

**Supplementary Material 3**

Supplementary Table 5. Longitudinal Regression Analyses Predicting Extreme Sleep Durations and Times-in-Bed

| **Model** | **Predictor** | **OR** | **CI** | **p** | **p (adjusted)** | **Fit Indices** |
| --- | --- | --- | --- | --- | --- | --- |
| Model 1 – Sleep Duration (<8 hours) | **Weight loss behaviours** | **.94**** | **.90-.98** | **p<.01** | **p<.01** | χ^2^(8, 5705)= 50.80, CFI=.98, TLI=.99, RMSEA=.03, SRMR=.03 |
|  | **Gender** | **1.10**** | **1.03-1.18** | **p<.01** | **p<.05** |  |
|  | BMI – cat | 1.02 | .95-1.09 | .65 | .74 |  |
|  | Household Income | .99 | .97-1.02 | .60 | .60 |  |
| Model 2 – Sleep Duration (>10 hours) | Weight loss behaviours | 1.03 | .96-1.11 | .39 | .52 | χ^2^(8, 5705)= 48.83, CFI=.99, TLI=.99, RMSEA=.03, SRMR=.02 |
|  | Gender | 1.07 | .94-1.21 | .30 | .30 |  |
|  | BMI – cat | .94 | .82-1.07 | .32 | .64 |  |
|  | **Household Income** | **.94**** | **.90-.98** | **p<.01** | **p<.05** |  |
| Model 3 – Time-in-Bed (<8 hours) | **Weight loss behaviours** | **.94*** | **.89-.99** | **p<.05** | **p<.05** | χ^2^(8, 5705)= 51.31, CFI=.98, TLI=.99, RMSEA=.03, SRMR=.03 |
|  | **Gender** | **.77***** | **.71-.84** | **p<.001** | **p<.001** |  |
|  | BMI – cat | 1.02 | .93-1.11 | .74 | .74 |  |
|  | Household Income | .99 | .96-1.02 | .48 | .60 |  |
| Model 4 – Time-in-Bed (>10 hours) | Weight loss behaviours | 1.03 | .94-1.12 | .52 | .52 | χ^2^(8, 5705)= 49.24, CFI=.99, TLI=.99, RMSEA=.03, SRMR=.02 |
|  | **Gender** | **1.17*** | **1.00-1.36** | **p<.05** | .06 |  |
|  | BMI – cat | 1.08 | .94-1.24 | .28 | .64 |  |
|  | **Household Income** | **.86***** | **.81-.91** | **p<.001** | **p<.001** |  |
| ***p<.001, **p<.01, *p<.05; for weight loss behaviours, lower values represent more weight loss behaviours | | | | | | |
